# Diagnostic accuracy of acyl-ghrelin and it association with non-alcoholic fatty liver disease in type 2 diabetic patients

**DOI:** 10.1186/s40200-015-0170-1

**Published:** 2015-05-19

**Authors:** Galyna Mykhalchyshyn, Nazarii Kobyliak, Petro Bodnar

**Affiliations:** Bogomolets National Medical University, T. Shevchenko blvd, 13, 01601 Kyiv, Ukraine

**Keywords:** Non-alcoholic fatty liver disease, Acyl-ghrelin, Type 2 diabetes

## Abstract

**Background:**

Ghrelin is a hormone produced mainly by the cells lining the fundus of the stomach, which is involved in regulation of lipid and glucose metabolism. Two major forms of ghrelin can be found in circulation: an acylated form, and non-acylated form. Serum acyl-ghrelin (AG) concentration is significantly increased in patients with visceral obesity and insulin resistance. This study was conducted to evaluate changes in serum AG levels, its diagnostic accuracy and association with NAFLD in patients with type two diabetes (T2D).

**Methods:**

In this cross-sectional study, 91 T2D patients, age of 40–80 years, were included. All patients were divided into 3 groups. The control group included 28 T2D patients without NAFLD. The main group included 63 T2D patients with NAFLD, which was divided in 2 subgroups depending on transaminase levels: normal (*n* = 37) and elevated (*n* = 26) transaminases group. To assess the diagnostic accuracy of AG for NAFLD we used ROC-analysis.

**Results:**

We observed 1.5 (*p* = 0.016) and 2.5 (*p* < 0.001) fold increasing of serum AG levels in patients with NAFLD and normal or elevated transaminases compared to control groups. In multivariate logistic regression analysis high AG level was an independent, from transaminases activity, triglycerides (OR 1.791; 95 % CI 1.162–2.759; *p* = 0.008) and degree of IR (OR 1.599; 95 % CI 1.019–2.508; *p* = 0.044) predictor that associated with NAFLD. When serum AG used as non-invasive marker for NAFLD detection AUROC was 0.835 (95 % CI 0.752–0.918, *p* < 0.001). The cut-off value was >0.52 ng/ml, with sensitivity, specificity, PPV and NPV – 60.3 %, 92.8 %, 95.0 %, 50.9 % respectively. For distinguishing patients with NAFLD and elevated transaminases from patients with NAFLD and normal values AG was less effective.

**Conclusions:**

Our study has demonstrated that elevated AG level were associated with NAFLD. Patients with elevated transaminases had significantly higher AG levels. An increase of AG over 0.52 ng/ml can be used as a diagnostic marker for NAFLD detection in patients with T2D.

## Background

Non-alcoholic fatty liver disease (NAFLD), the hepatic counterpart of the metabolic syndrome (MS) [1], encompasses a disease spectrum spanning simple steatosis through nonalcoholic steatohepatitis (NASH) with/without cirrhosis, and hepatocellular carcinoma [2]. The obesity and type 2 diabetes (T2D) pandemic and the improved management of chronic viral hepatitis, have resulted in NAFLD becoming a leading cause of chronic liver disease [3, 4] and a major health concern owing to hepatic and extrahepatic morbidity/mortality [5].

NAFLD pathogenesis as a two-hit model was initially proposed by Day and James [6]. First, IR causes lipid accumulation in hepatocytes and lead to development of fatty liver; second, cellular insults such as oxidative stress, lipid oxidation, and chronic inflammation result in NASH. In patients with NAFLD, the intrahepatic TG content seems to depend mainly on the systemic availability of free fatty acids (FFA). This was suggested by isotope labeling studies in which serum FFA were shown to account for 59 % of hepatic TG [7]. Therefore fatty liver develops when *de novo* synthesis exceed the oxidation and re-secretion of TGs. There are several mechanism which lead to this: (1) increased FFA supply due to increased lipolysis from both visceral/subcutaneous adipose tissue as a result of IR [8]; (2) increased intake of dietary fat sources of FFAs; (3) increased *de novo* lipogenesis [9]; (4) alteration in the synthesis or secretion of lipoproteins [10]; (5) reduction in mitochondrial FA β-oxidation [11]. There is evidence that rate of *de novo* lipid synthesis is elevated in livers of patients with NAFLD, compared with healthy subjects [12]. A shift from FA oxidation to *de novo* lipid synthesis is mediated by an increased activity of the transcription factors PPAR-γ [13], ChREBP and SREBP-1c [14], all of which are positive modulators of hepatic TG contents by targeting genes coding for key reactions in lipid synthesis.

Ghrelin is a 28 amino-acid peptide with an n-octanoyl group at the serine three residue, produced mainly by the stomach, which was identified as the endogenous ligand of the growth hormone secretagogue receptor (GHS-R) [15]. Administration of exogenous ghrelin reportedly enhances appetite and increases food intake through the activation of hypothalamic neuropeptide Y/agouti-related peptide neurones expressing GHS-R type 1a [16]. In addition to potent GH-releasing activity, ghrelin is involved in the peripheral levels influencing lipid metabolism [17] and glucose homeostasis by regulating insulin secretion and sensitivity in pancreatic b-cells [18] and by stimulating glucose output by primary hepatocytes [19].

The preproghrelin gene-derived peptides include acyl ghrelin (AG), DAG, and obestatin [1]. Earlier studies on ghrelin and energy balance and metabolism were performed by total ghrelin assays. It was decreased in obesity, insulin resistance (IR) and T2D [16, 19]. DAG is the major circulating form and constitutes 80–90 % of circulating ghrelin. Although it was originally thought that DAG lacked endocrine and biological actions, more recent findings suggest that both AG and DAG may mediate peripheral biological actions; indeed, there is a suggestion that both may act antagonistically [20, 21]. Therefore, the aim of this study was to explore the potential role of AG in NAFLD and to assess it diagnostic accuracy as non-invasive marker.

## Materials and methods

### Study subjects

In this cross-sectional study, 112 T2D patients with age of 40–80 years from the Kyiv City Clinical Endocrinology Center were selected. Inclusion criteria were: age over 18 years, presence of T2D in association with or without NAFLD. NAFLD diagnosis was concluded according to the recommendations of the American Gastroenterology Association (AGA) and American Association for the Study of Liver Disease (AASLD) on the basis of next: clinical examination, laboratory values of lipid and carbohydrate metabolism, liver enzyme activities (ALT, AST), ALT/AST ratio, and ultrasonography (US) examination [22]. Exclusion criteria included alcohol abuse (>210 grams of alcohol per week in men and >140 g of alcohol per week in women over a two-year period), chronic viral hepatitis (associated with HBV, HCV, HDV infection), drug-induced liver disease, Wilson’s disease, hereditary deficiency of antitrypsin-1 and idiopathic hemochromatosis. We met criteria of eligibility in 91 T2D patients which were included in this study. In 21 patients we found comorbid etiology with signs of others chronic liver disease: 15 of them were seropositive for chronic viral hepatitis, 6 patients have alcohol abuse anamnesis. These patients were excluded from our study. The ethics committee of Kyiv City Clinical Endocrinology Center approved the study.

### Data collection and measurements

After informed consent, fasting serum samples were obtained and immediately frozen at−80 °C. For each patient, relevant clinical and demographic data were collected. Anthropometric data including weight and height were measured to the nearest 100 g and 0.5 cm, respectively. Body mass index (BMI) was calculated as body weight in kilograms divided by the square of the participant’s height in meters.

Plasma total cholesterol (TC), HDL-cholesterol (HDL-C) and triglyceride (TG) concentrations were measured using enzymatic kits, standardized reagents and standards (BioVendor, Czech Republic). LDL-cholesterol concentration was calculated using the Friedewald equation [23]. Blood glucose was determined using the Trinder’s glucose oxidase method while serum insulin was measured with the double radioimmunoassay (RIA) method (AIA-Pack IRI; Tosoh, Tokyo). Insulin resistance was assessed by the validated homeostasis model assessment (HOMA) index [24] using the following formula: HOMA-IR = (FPG * FPI)/22.5, where FPG and FPI are fasting plasma glucose (mmol) and fasting plasma insulin (μU/ml), respectively.

The diagnosis of fatty liver was based on the results of abdominal ultrasonography, which was done by trained technicians with Ultima PA (Radmir Co., Kharkiv, Ukraine). Of 4 known criteria (hepatorenal echo contrast, liver brightness, deep attenuation, and vascular blurring) [25], the participants were required to have hepatorenal contrast and liver brightness to be given a diagnosis of NAFLD.

Also for confirmation of NAFLD diagnosis especially in case of US negative or contradictory data we used computer-assisted quantitative US techniques assessment. In these cases we used software programs to analyze US echo attenuation or calculate hepato-renal index which defined as the ratio of the echo intensities of the liver and renal cortex. These parameters have demonstrated higher sensitivities (>90 %) and specificity ((>90 %)) for mild steatosis detection (≥5 %) as compared to routine B-mode US [26, 27].

Fasting serum AG concentrations were measured from plasma samples stored at −20 °C using a commercial “peptide enzyme immunoassay” kit (Peninsula Laboratories, Inc., California, USA) with intra-assay and inter-assay variations of coefficients (CV) of less than 5 % and 14 %, respectively, sensitivity limit of 0.08-1.0 ng/ml, and measuring range of 0–25 ng/ml. No cross-reactivity was found with human des-acyl ghrelin, motilin, secretin, human vasoactive intestinal peptide, human prolactin-releasing peptide-31, human galanin, human growth hormone releasing hormone, neuropeptide Y, orexin A, orexin B.

### Statistical analysis

The SPSS statistical package, version 20.0 (SPSS, Inc., Chicago, Illinois) was used for all statistical analyses and a *P* value less than 0.05 was considered statistically significant. All continuous values are expressed as mean ± SD and categorical variables are presented as %. Data distribution was analyzed using the Kolmogorov-Smirnov normality test. Continuous variables with parametric distribution were then analyzed using Analysis of Variance (ANOVA) and if the results were significant, a Bonferroni Post Hoc test was performed. Data with non-parametric distribution was analyzed using Kruskall-Wallis test. For comparisons of categorical variables we conducted *χ*^2^ test.

Univariate and multivariate logistic regression analyses were used to identify the risk factors of NAFLD. The odds ratios are given with the 95 % confidence intervals. Variables which are statistically significant in univariate analysis were included in the multivariate logistic regression analysis. Backward stepwise selection was used at a stringency level of *p* < 0.10 to detect the independent risk factors on NAFLD. A *p* < 0.05 probability level was considered as statistically significant.

To assess the diagnostic accuracy of AG for NAFLD detection and differentiation patients with normal and elevated transaminases values we used receiver operating characteristic (ROC) curves. The ROC curve is a plot of sensitivity (Se) *vs* 1-specificity (Sp) for all possible cut-off values. The most commonly used index of accuracy is area under the ROC curve (AUROC). AUROC-values close to 1.0 indicated high diagnostic accuracy. Optimal cut-off values were chosen to maximize the sum of sensitivity and specificity, and positive (PPV) and negative predictive values (NPV) were computed for these cut-off values.

## Results

All patients were divided on the next groups. The control group included 28 (30.76 %) T2days patients without NAFLD (mean age was 53.57 ± 7.16, T2D duration 3.5 ± 1.57). The main group included 63 (69.24 %) T2D patients with NAFLD, which was divided in 2 subgroups depending on transaminase levels: normal transaminases group included 37 (58.73 %) patients (mean age was 53.27 ± 8.39, T2D duration 5.97 ± 3.88 years) and respectively elevated transaminases group included 26 (41.27 %) patients with mean age 51.5 ± 10.92 and T2D duration 8.54 ± 5.57 years.

Results of anthropometric, clinical and laboratory parameters are represented in the Table 1. There was no statistically significant difference in the T2D duration between all study groups (*p* = 0.068). The longest duration of T2D was observed in patients with NAFLD and elevated transaminases. BMI was elevated in all patients. We found significant increasing of BMI parallel to NAFLD presence and it severity. The prevalence of obesity (defined as BMI>30.0) in control group was 64.3 %, while in NAFLD patients the rate were higher: 75.7 % for normal transaminases and 100 % for elevated transaminases group (*p* = 0.004) respectively. Morbid obesity (BMI>40.0) was diagnosed only in patients with NAFLD (16.2 % vs 46.2 %, *p* < 0.001).

**Table 1:** Anthropometric, clinical and laboratory parameters in examined patients (M ± SD)

Parameters	Control	p1	p2	NAFLD with normal transaminases	NAFLD with elevated transaminases	p3	p
	(n = 28)			(n = 37)	(n = 26)		
Age, years	53.57 ± 7.16	-	-	53.27 ± 8.39	51.5 ± 10.92	-	NS
Duration of T2D, years	5.0 ± 2.81	-	-	5.97 ± 3.88	8.54 ± 5.57	-	NS
BMI, kg	31.15 ± 3.0	0.028	<0.001	34.46 ± 5.64	40.35 ± 5.63	<0.001	<0.001
ALT, U	25.03 ± 7.26	NS	<0.001	28.93 ± 6.15	64.96 ± 14.99	<0.001	<0.001
AST, U/L	23.91 ± 6.11	NS	<0.001	26.6 ± 5.71	55.8 ± 11.91	<0.001	<0.001
FPI, μIU/ml	11.21 ± 3.58	0.001	<0.001	18.27 ± 8.14	20.61 ± 8.56	NS	<0.001
FPG, mmol/l	7.80 ± 1.41	-	-	8.67 ± 3.25	9.48 ± 3.14	-	NS
HOMA-IR	3.86 ± 1.41	<0.001	<0.001	6,86 ± 3.21	8.23 ± 2.85	NS	<0.001
TC, mmol/l	5.68 ± 0.68	0.004	<0.001	6.24 ± 0.72	6.56 ± 0.62	NS	<0.001
TG, mmol/l	1.83 ± 0,45	0,018	<0,001	2,65 ± 1,2	3,36 ± 1,55	NS	<0,001
VLDL-C, mmol/l	0.82 ± 0.2	0.005	<0.001	1.25 ± 0.59	1.39 ± 0.63	NS	<0.001
HDL-C, mmol/l	1.66 ± 0.25	0.001	<0.001	1.43 ± 0.24	1.2 ± 0.26	0.002	<0.001
LDL-C, mmol/l	3.18 ± 0.6	0.047	<0.001	3.61 ± 0.69	4.07 ± 0.78	0.034	<0.001

p - the difference between all study groups calculated using one-way ANOVA

p 1-3 - for pairwise comparisons used Bonferroni Posthoc test

p1 - the difference between the control and normal transaminases groups

p2 - the difference between the control and elevated transaminases groups

p3 - the difference between normal and elevated transaminases groups

In all patients we found violation of the carbohydrate metabolism. Mean values of HOMA-IR and insulinemia were higher in NAFLD patients with maximum on elevated transaminases groups (*p* < 0.001). The value of HOMA-IR exceeding 3 (indication of IR) were found in 78.6 % of patients from control group, in NAFLD groups, depending of transaminase activity, we determine IR in 94.6 % and 96.2 % patients respectively (*p* = 0.048).

Dyslipidemia was present in all study patients. We observed growth of TC (*p* < 0.001), TG (*p* < 0.001), VLDL-C (*p* < 0.001) and LDL-C (*p* < 0.001) parallel with the development of NAFLD and activity of transaminase. While HDL-C levels decreased. Highest mean lipid levels were observed in patients with NAFLD and elevated transaminases.

**Fig. 1:**
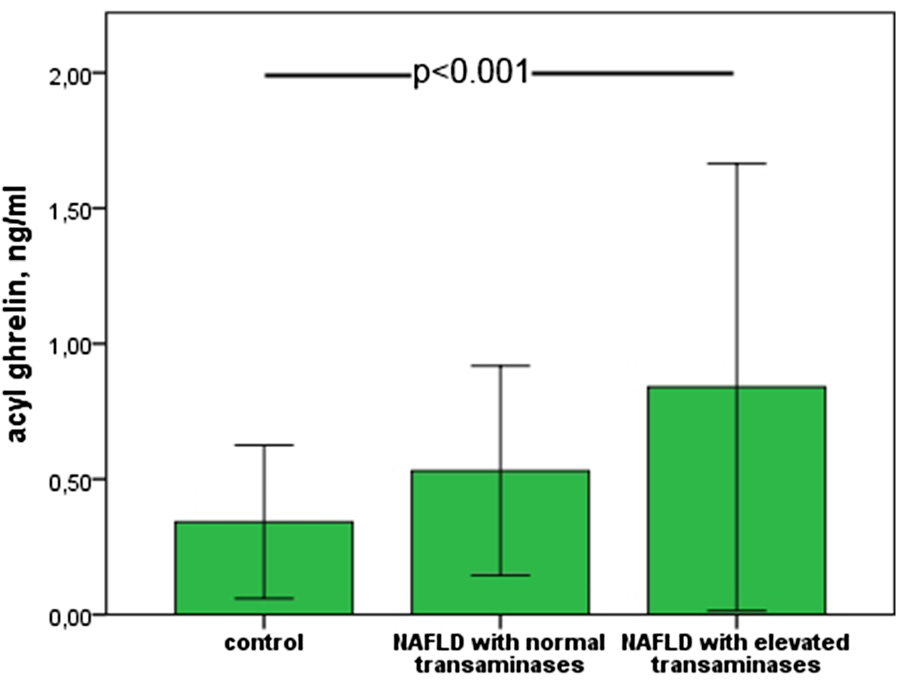
Serum AG levels in study patients

Level of serum AG was significantly higher in patients with NAFLD compared to the control group (Fig. 1). We also mentioned significant difference between mean AG values in NAFLD patients depending on transaminase activity (0.53 ± 0.19 vs 0.83 ± 0.41, *p* = 0.001).

In univariate logistic regression analysis independent predictors, associated with the development NAFLD in patients with T2D were HOMA-IR (OR 2.026; 95 % CI 1.409–2.914; *p* < 0.001), insulin (OR 1,307; 95 % CI 1.140–1.500; *p* < 0.001), BMI (OR 1.296; 95 % CI 1.130–1.436; *p* < 0.001), ALT (OR 1.121; 95 % CI 1.051–1.196; *p*–0.001) and AST (OR 1.128; 95 % CI 1.052–1.196, *p*–0.001). The level of AG was also independently associated with NAFLD development (OR 2.104; 95 % CI 1.465–3.021; *p* < 0.001).

All variables which were statistically significant in univariate analysis were included in the stepwise multivariate logistic regression analysis. As seen in Table 2 we constructed several regression models. From which AG was an independently from transaminases activity, level of TG (Nagelkerke R^2^ = 0,624) and degree of IR (Nagelkerke R^2^ = 0,529) associated with the NAFLD development.

**Table 2:** Multiple stepwise logistic regression analysis using NAFLD as dependent variable and as independent predictors all factors that were significant associated in univariate analysis

Models	Regression coefficient ± SE	OR (95 % CІ)	p
Model 1 (Nagelkerke R^2^ = 0,624)			
Constant	-8.971 ± 2.32		
Acyl-ghrelin	0.583 ± 0.221	1.791 (1.162–2.759)	0,008
ALT	0,116 ± 0.046	1.123 (1.026–1.230)	0,012
TG	1.811 ± 0.701	6.119 (1.548–24.183)	0.010
Model 2 (Nagelkerke R^2^ = 0,569)			
Constant	-5.984 ± 1.675		
Acyl-ghrelin	0.469 ± 0.230	1.599 (1.019–2.508)	0.041
HOMA-IR	0.386 ± 0.191	1.471 (1.010–2.140)	0.044
AST	0.099 ± 0.046	1.104 (1.008–1.208)	0.033

**Fig. 2:**
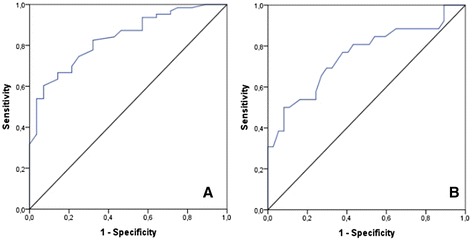
ROC-curves using as a predictor AG for NAFLD diagnosis (**a**) and distinguish patients with different degree of transaminase activity (**b**)

Establishing a statistically significant difference between mean AG levels in patients with and without NAFLD we created a background for assessment of diagnostic efficacy of this hormone in diagnosing NAFLD. In these purposes, we have used ROC-analysis. We have plotted 2 ROC-curves (Fig. 2).

The first ROC-curve was used to analyze AG as a predictor for NAFLD diagnosis. In this model we included overall data from patients with NAFLD independent of transaminase activity and compared it to subjects with T2D without NAFLD. We determined the quality of this diagnostic model as very good since AUROC for AG was 0.835 (95 % CI 0.752–0.918, *p* < 0.001) (Fig. 2, Table 3). The cut-off value of AG for NAFLD diagnosing in patients with T2D was >0.52 ng/ml. Sensitivity, specificity, PPV and NPV were 60.3 %, 92.8 %, 95.0 %, 50.9 % respectively.

**Table 3:** Diagnostic accuraccy of AG for NAFLD diagnosis and differentiation between patients with elevated and normal values

Parameter	NAFLD vs Control	NAFLD (normal vs elevated transaminases)
Cut-off value	<0.52	<0,84
Sensitivity, %	60.3	50.0
Specificity, %	92.8	91.8
NPV, %	50.9	72.3
PPV, %	95.0	81.2
AUROC	0.835	0.753
95 % CІ	0.752–0.918	0.625–0.880
p (AUROC)	<0,001	0.001

*NPV* negative predictive value, *РPV* positive predictive value, *AUROC* area under ROC-curve, *95%CІ* 95 % confidence interval for AUROC

The 2^nd^ ROC-curve was constructed to analyze AG efficacy in differentiating patients with NAFLD and normal or elevated transaminases. The quality of this diagnostic model was describes as poor, since AUROC for ghrelin was 0.753 (95 CI 0.625–0.880, *p* = 0.001). The cut-off value for ghrelin for diagnosing elevated transaminases in patients with NAFLD and T2D was >0.84 ng/ml. Sensitivity, specificity, PPV and NPV were 50.0 %, 91.8 %, 81.2 %, 72.3 % respectively (Table 3).

## Discussion

NAFLD is characterized by accumulation of TG in the hepatocytes as well as other inflammatory and fibrotic changes in the hepatic parenchyma. Our study assesses the potential connections between NAFLD and serum AG concentrations in T2D patients. The first available data mentioned a negative association between total ghrelin and NAFLD. Marchesini et al. when compared to 40 matched healthy subjects, suggested that patients with NAFLD (*n* = 86) had reduced level of total ghrelin. After adjustment for age and sex, significantly associated with low ghrelin (below 235pmol/l) in the whole group was only HOMA-IR (OR 5.79; 95 % CI 2.62–12.81; *p* < 0.0001) [28]. Tacke et al. reported that total ghrelin levels are elevated in elevated in Child C cirrhosis patients independent of the etiology of chronic liver diseases. Ghrelin did not correlate with liver function, but associated with clinical (gastrointestinal bleeding, ascites, encephalopathy) and biochemical (anemia, inflammatory markers, hypoglycaemia, renal dysfunction) parameters [29].

Therefore role of different ghrelin forms are still contradictory. We reported 1.5 (*p* = 0.016) and 2.5 (*p* < 0.001) fold increasing of serum AG levels in patients with NAFLD and normal or elevated transaminases compared to control groups. Our data in concordance with the study of Estep et al [30]. They reported, in 75 morbidly obese patients with biopsy-proven NAFLD (41 with histologic NASH), that circulating AG concentrations in NASH with fibrosis score ≥2 were almost double the concentration of NASH patients with a fibrosis stage <2 (8.73 vs. 4.22 pg/ml, *p* < 0.04). However no significant differences in AG levels between NASH and patients with non-NASH (6.42 ± 15.0 vs 2.85 ± 6.0, p>0.05) authors did not found. From the other hand Gutierrez-Grobe et al. [31], in cross-sectional study included 98 subjects (51 NAFLD patients and 47 controls), found that compared to control NAFLD patients had significantly lower level of serum ghrelin. In multivariate logistic regression analyses high ghrelin (OR = 0.128, 95 CI 0.048–0.343, *p* < 0.001) level had a protective effect against hepatic steatosis after controlling for potential confounders. The limitations of this study were lack of liver biopsy and image-based NAFLD diagnosis. Also the authors used an assay without using protease inhibitors that are essential to protect AG from being de-acylated, which questions what peptides are really involved in their observed associations [32]. Due to our data high AG level was an independent, from transaminases activity, TG (OR 1.791; 95 % CI 1.162–2.759; *p* = 0.008) and degree of IR (OR 1.599; 95 % CI 1.019–2.508; *p* = 0.044) predictor that associated with NAFLD development in patients with T2D.

Recent study showed that incubation with both AG and DAG, in differentiating omental adipocytes, significantly increased PPAR-γ and SREBP1 mRNA levels, as well as several fat storage–related proteins, including acetyl-CoA carboxylase (ACC), fatty acid synthase (FAS), lipoprotein lipase (LPL) and perilipin [33]. Consequently, both the ghrelin forms may play a role in excessive fat accumulation in obesity and thereby NAFLD.

Also in state of NAFLD elevated concentrations and systemic availability FFA may promote increased ghrelin acylation which recognized as one of putative mechanisms for the elevated circulating AG [34]. Nishi et al. report that ingestion of either medium-chain fatty acids (MCFAs) or medium-chain triacylglycerols (MCTs) increased the stomach concentrations of AG without changing the total (acyl- and des-acyl) ghrelin amounts in mice [35]. Interestingly, Yang et al. [36] have recently identified a novel enzyme implicated in the n-octanoylation of ghrelin, namely GOAT (Ghelin O-Acyltransferase), that is expressed in the major ghrelin-secreting tissues. It seems plausible that obesity influences the expression and/or activity of this acyltransferase, leading to elevated plasma concentrations of AG.

## Conclusion

In conclusion, our data indicate that relative AG excess is associated with NAFLD in T2D patients independently of IR, transaminases activity and TG levels. We first provided the assessment of diagnostic accuracy of AG for NAFLD diagnosis and distinguishing patients with different degree of transaminase activity and concluded high specificity (>90 %) with lower value of sensitivity (50–60 %). Furthermore investigations will be needed to elucidate whether dysregulation of ghrelin secretion profiles in NAFLD patients may influence the long-term metabolic outcomes.

## Abbreviations

AGA: American Gastroenterology Association
AASLD: American Association for the Study of Liver Disease
AG: Acyl-ghrelin
ALT: Alanine transaminase
ANOVA: Analysis of variance
AST: Aspartate transaminase
AUROC: Area under the ROC curve
BMI: Body mass index
DAG: Dys-acyl ghrelin
FFA: Free fatty acids
FPG: Fasting plasma glucose
FPI: Fasting plasma insulin
HDL-C: High density lipoprotein
HOMA: Homeostasis model assessment
LDL: Low density lipoprotein
NAFLD: Non-alcoholic fatty liver disease
NASH: Non-alcoholic steatohepatitis
NPV: Negative predictive values
PPV: Positive predictive values
ROC: Receiver operating characteristic
Se: Sensitivity
Sp: Specificity
TC: Total cholesterol
TG: Triglyceride
VLDL-C: Cholesterol of very low density lipoproteins
US: ultrasonography
